# Ferro-piezoelectricity in emerging Janus monolayer BMX_2_ (M = Ga, In and X = S, Se): *ab initio* investigations

**DOI:** 10.1039/d2na00597b

**Published:** 2023-01-26

**Authors:** Djamel Bezzerga, El-Abed Haidar, Catherine Stampfl, Ali Mir, Mohammed Sahnoun

**Affiliations:** a Department of Physics, Ahmed Zabana University of Relizane Algeria bezzergadjamel@yahoo.fr; b School of Physics, The University of Sydney New South Wales 2006 Australia ehai2584@uni.sydney.edu.au; c Laboratory of Quantum Physics of Matter and Mathematical Modeling (LPQ3M), University Mustapha Stambouli of Mascara Algeria; d Department of Physics, Dr Tahar Moulay University of Saida Algeria

## Abstract

Nanoscale materials with inter-correlation characteristics are fundamental for developing high performance devices and applications. Hence theoretical research into unprecedented two-dimensional (2D) materials is crucial for improving understanding, especially when piezoelectricity is merged with other unique properties such as ferroelectricity. In this work, an unexplored 2D Janus family BMX_2_ (M = Ga, In and X = S, Se) corresponding to group-III ternary chalcogenides has been explored. The structural and mechanical stability, and optical and ferro-piezoelectric properties of BMX_2_ monolayers were investigated using first-principles calculations. We found that the lack of imaginary phonon frequencies in the phonon dispersion curves establishes the dynamic stability of the compounds. The monolayers BGaS_2_ and BGaSe_2_ are indirect semiconductors with bandgaps of 2.13 eV and 1.63 eV, respectively, while BInS_2_ is a direct semiconductor with a bandgap of 1.21 eV. BInSe_2_ is a novel zero-gap ferroelectric material with quadratic energy dispersion. All monolayers exhibit a high spontaneous polarization. The optical characteristics of the BInSe_2_ monolayer show high light absorption ranging from the infrared to the ultraviolet. The BMX_2_ structures exhibit in-plane and out-of-plane piezoelectric coefficients of up to 4.35 pm V^−1^ and 0.32 pm V^−1^. According to our findings, 2D Janus monolayer materials are a promising choice for piezoelectric devices.

## Abstract 2

Nanoscale materials with inter-correlation characteristics are fundamental for developing high performance devices and applications.

## Graphical abstract

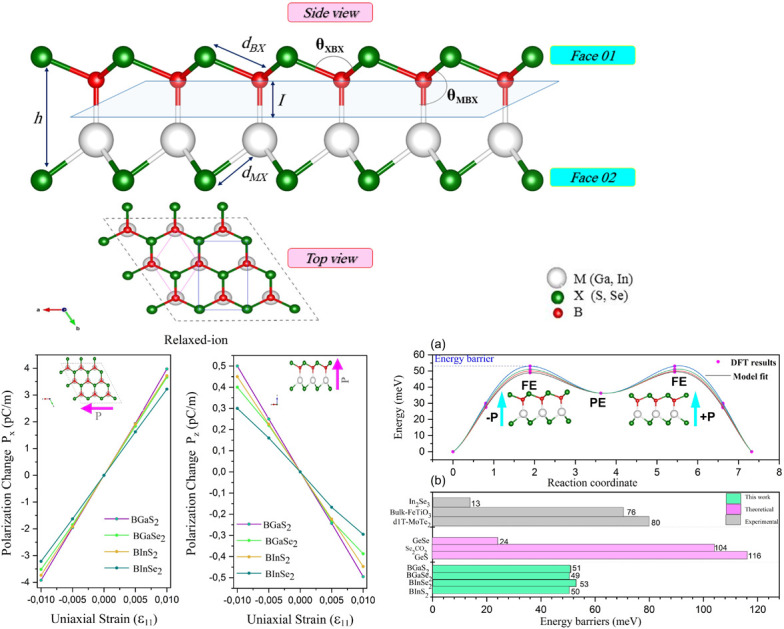

## 1 Introduction

The discovery of graphene in 2004 has led scientists from various fields to investigate two-dimensional (2D) materials such as hexagonal boron nitride (h-BN), transition metal chalcogenides (TMDs), 2D Xenes and more.^1^ Such a variety of 2D materials is attractive due to their desirable mechanical, physical, structural and chemical properties, which benefit applications such as electronics, sensors and batteries.^2^ Recently, there has been great interest in the synthesis of 2D materials with structures containing single or binary elements. Binary 2D materials can be formed by stacking two single-element layers of different compositions, such as MoS_2_/WS_2_, or by chemically interweaving two single-element layers, such as MoTe_2_. These heterostructures can exhibit novel properties that are not found in the constituent layers, such as enhanced electrical conductivity, improved thermal conductivity, and novel optical properties. However, more recently the focus has shifted to unusual 2D ternary materials because of their unique properties arising when a third element is added. Unlike phosphorene or boron nitride (binary), the extra degree of freedom in 2D ternary materials can adjust their properties by modifying the compositions, which can lead to novel device applications.^3^

A recent unconventional 2D family of structures, known as 2D Janus materials, rely on having out-of-plane structural symmetry.^4^ Tailoring 2D Janus layers requires having different atoms on each side of the 2D layer. A major experimental breakthrough was the synthesis of transition metal di-chalcogenide (TMD) Janus monolayers where the molybdenum disulfide MoS_2_ structure, which involves two layers of sulfur (S) sandwiching a molybdenum (Mo) layer, has replaced one of its S layers by a selenium (Se) layer.^5^ Using first-principles calculations, Nandi *et al.*^6^ explored Janus MXY (M = Ge, Sn, and X/Y = S/Se) structures and found that GeSSe monolayers and bilayers are energetically favorable and stable with high shear and in-plane piezoelectricity and high carrier mobility and are flexible. On the basis of *ab initio* calculations, Demirtas *et al.*^7^ investigated aluminum monochalcogenide Janus Al_2_XX′ (X/X′: O, S, Se, Te) monolayers and found that applying biaxial compressive or tensile strain can possibly lead to indirect–direct band-gap transitions. Also using *ab initio* calculations, Varjovi *et al.*^8^ demonstrated the dynamical and thermal stability of Janus WXO (X = S, Se, and Te) monolayers, which were found to exhibit orientation dependent mechanical properties, a band gap that narrows along the chalcogen group, and large-piezoelectric properties. Experimentally, Fu *et al.*^9^ have shown how the fabrication of Sr_0.9_Ce_0.05_Fe_0.95_Ru_0.05_O_3_ provided a potential heterostructure electrocatalyst when embedded into *in situ* grown RuO_2_. Captivating properties such as a second-harmonic generation (SHG) response,^10^ strong Rashba spin splitting,^11^ efficient catalytic performance^12^ and a significant piezoelectric effect have been achieved due to quantum confinement effects that arise at the nanoscale, unlike in the bulk form.^13^ Such properties have high potential for engineering applications such as sensors, actuators and novel electro-mechanical devices.^14^

The dual properties of 2D piezoelectricity and ferroelectricity are very exciting and such structures have been confirmed, within a single monolayer, experimentally and predicted theoretically.^15^ Piezoelectricity^13^ involves the conversion between electrical and mechanical energy, while ferroelectricity involves having spontaneous polarization which can be reversed by an external electric field.^16^ Piezoelectricity was firstly theoretically predicted in the 1H phase of monolayer MoS_2_ by Duerloo *et al.*^17^ in 2012 and was experimentally verified in 2014 by Wu *et al.*,^18^ where increasing the number of layers led to minimizing the piezoelectric coefficient. It is desirable to investigate other semiconductors with a high piezoelectric coefficient for efficient inter-conversion of nano-electro-mechanical energy, whilst being a semiconductor benefits nanoelectronic applications. High values of piezoelectric parameters have been reported for group-IV and group-VI transition-metal di-chalcogenide and di-oxide (TMDC and TMDO) monolayers.^19^ As for 2D ferroelectrics, In_2_Se_3_ and SnS have shown in-plane and out-of-plane ferroelectric polarization, which is almost impossible to be found in three-dimensional (3D) ferroelectrics.^20^ 2D ferroelectric materials also exhibit other exciting properties such as piezoelectric and photovoltaic effects, and valley and spin polarization that could benefit functionalities such as tunnel junction field-effect transistors and photodetectors.^21^ An example is In_2_Se_3_ which is a 2D ferroelectric semiconductor with low voltage. This structure was experimentally shown to have a high memory window and on/off ratio which is key for applications as a ferroelectric field-effect transistor.^22^ This report encourages further investigation into the piezoelectric and ferroelectric properties of 2D materials.

In this study, we theoretically construct four BMX_2_ monolayers: BGaS_2_, BGaSe_2_, BInS_2_ and BInSe_2_. We investigate their stability and corresponding properties using density functional theory (DFT). Our calculations indicate that all of the structures discussed are dynamically stable and exhibit promising optoelectronic and piezoelectric characteristics when compared to previously investigated 2D piezoelectric materials.^23,24^ The paper is organized as follows: in Section 2, the computational methodology is given, and in the following Results and discussion section, we present the structural, mechanical, optoelectronic and ferro-piezoelectric properties of the BMX_2_ monolayers. Finally, we give the conclusion.

## 2 Computational methodology

The first-principles calculations based on DFT were performed using with the Quantum-ESPRESSO^25^ code. The exchange-correlation functional utilized was the Perdew–Burke–Ernzerhof (PBE)^26^ generalized gradient approximation (GGA).^27^ The ion cores were treated using projector augmented-wave (PAW) pseudopotentials with a cut off energy of 70 Ry. The charge density was expanded in a basis set with a 280 Ry plane wave cutoff. Brillouin zone sampling was done using a Monkhorst Pack mesh of 14 × 14 × 1 ***k***-points.^28^ The cutoff for the structural and ionic relaxation is taken to be when the force on each atom is less than 10^−4^ Ry bohr^−1^. The total energy is regarded as being converged when the total energy difference between two consecutive steps of the self-consistent electronic cycle is less than 10^−8^ Ry. To avoid interactions between periodically repeated unit cells, the vacuum region is set to 20 Å. Since the GGA underestimates bandgaps, we employed the HSE06 hybrid functional^29^ for more accurate bandgap calculations. The dynamic stability for each 2D Janus layer was studied. The piezoelectricity calculations used density functional perturbation theory (DFPT).^30^ From the dynamic matrix, the phonon dispersion curves were obtained to find the calculated second-order force constants. A supercell of 3 × 3 × 1 and a 4 × 4 × 1 *k*-point sampling were used for all the studied compounds. To calculate the piezoelectric coefficients and ferroelectric properties (*i.e.* electric spontaneous polarization), we employed the Berry Phase method as implemented in the Quantum-ESPRESSO package,^31^ which has been extensively discussed in earlier publications.^32–34^ To calculate the optical properties and elastic constants *C*_*ij*_, the plane-wave code themo_pw was used.^25^ Plots of the atomic structures, bond angles, and bond lengths were produced using Vesta software.^35^

## 3 Results and discussion

### 3.1 Structural stability

**Fig. 1:**
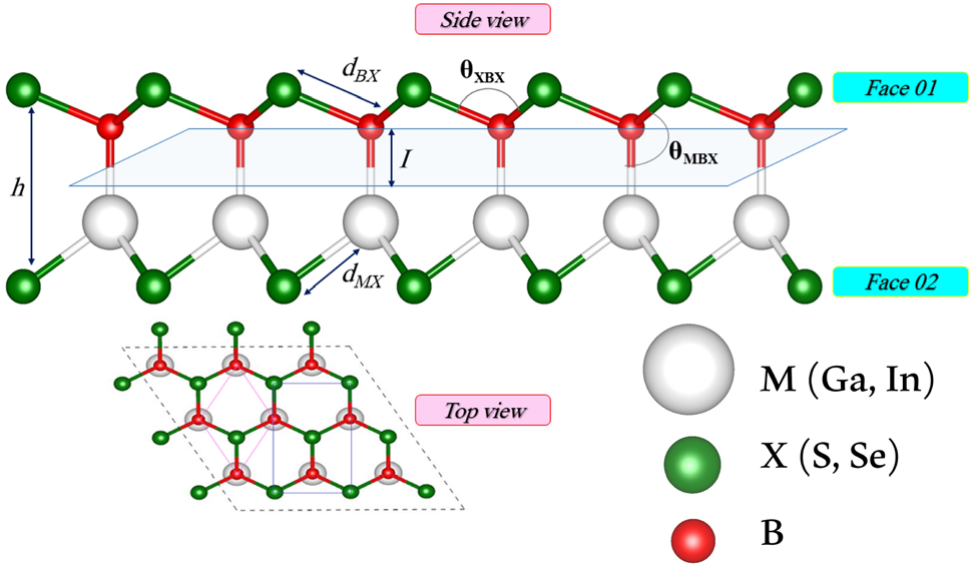
Side and top views of the optimized 2D Janus monolayer BMX_2_ compounds, where B and M are shown in red and white, and X (S and Se) atoms are shown as green spheres, respectively.

We first investigate the geometric optimization of the four 2D Janus monolayers BGaS_2_, BGaSe_2_, BInS_2_ and BInSe_2_ which are structurally similar. All four structures contain 4 atoms in the unit cell; one of the atoms is M type (Ga or In), and one of the atoms is a B atom, both of which are sandwiched between X (S or Se) atoms which are located half above and half below the M–B atoms. Each boron atom is covalently bonded to three chalcogenide X atoms and one M atom, resulting in a trigonal prismatic geometry with space group no. 187 (*P*6̄*m*2). To construct the Janus structure of BSe for instance, the bottom B atoms are substituted by In atoms, forming a BInSe_2_ Janus monolayer. This structure is formed by connecting the first face layer BX (BSe) to the second face layer MX (InSe) in such a way that a bridge bond is formed between the boron and indium atoms (see Fig. 1). The BMX_2_ structure, in the equilibrium state, belongs to the *C*_3v_ point group symmetry with polar space group no. 156 (*P*3*m*1). Because of the BX and MX *D*_3h_ symmetry, the replacement of B atoms by M atoms broke the out-of-plane structure symmetry. Furthermore, the difference in the electronegativity and atomic number of B, M and X atoms causes in-equivalent B–X and M–X bond lengths and in-plane lattice constants in these Janus monolayers. These as well as other structural parameters are tabulated in Table 1 along with the band gap energies. The optimized lattice constant of monolayer BInSe_2_ of 3.59 Å is larger than that of the other BMX_2_ monolayers (3.29–3.46 Å). Furthermore, the bond lengths of B–X and M–X are not equivalent due to the difference in the atomic radius and electronegativity of the atoms. To evaluate the energetic stability, we calculated the cohesive energy with the following formula:1
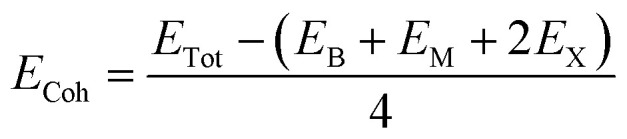
where *E*_Tot_ is the total energy of the Janus BMX_2_ monolayers; *E*_B_, *E*_M_, and *E*_X_ are the energies of boron, M, and X atoms in their solid phases, “2” is the numbers of X atoms and “4” is the total number atoms in the unit cell. The cohesive energy gives insight into the strength of the covalent bonds in the BMX_2_ monolayers. The negative values for the cohesive energy indicate that the formation of such monolayers is an exothermic process.^36^

**Table 1:** Optimized structural parameters and band gap energies (*E*_g_) of 2D Janus BMX_2_ compounds: lattice constant (*a*), bond length (*L*) between group-III atoms, distance (*h*) between chalcogen atoms (*i.e.*, layer thickness), bond length (*d*) between chalcogen (X = S, Se) and group-III (M = Ga, In) atoms, bond angles 
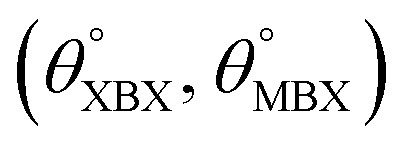
, the cohesive energies (*E*_Coh_) and the energy band-gap (*E*_g_) in PBE and HSE06. See Fig. 1 for the corresponding geometric information

Property	*a* (Å)	*L* (Å)	*h* (Å)	*d*_BX_ (Å)	*d*_MX_ (Å)	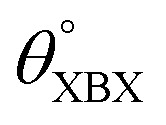	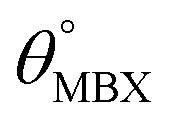	*E*_Coh_ (eV)	*E*_g_ (eV_PBE_)	*E*_g_ (eV_HSE06_)
BGaS_2_	3.29	2.08	4.11	2.06	2.26	105.99	112.75	−5.02	1.33	2.13
BGaSe_2_	3.46	2.07	4.27	2.19	2.39	104.64	113.95	−4.85	0.92	1.63
BInS_2_	3.40	2.29	4.55	2.12	2.45	107.02	111.82	−4.57	0.42	1.21
BInSe_2_	3.59	2.28	4.70	2.25	2.58	105.75	112.97	−4.23	0	0

**Fig. 2:**
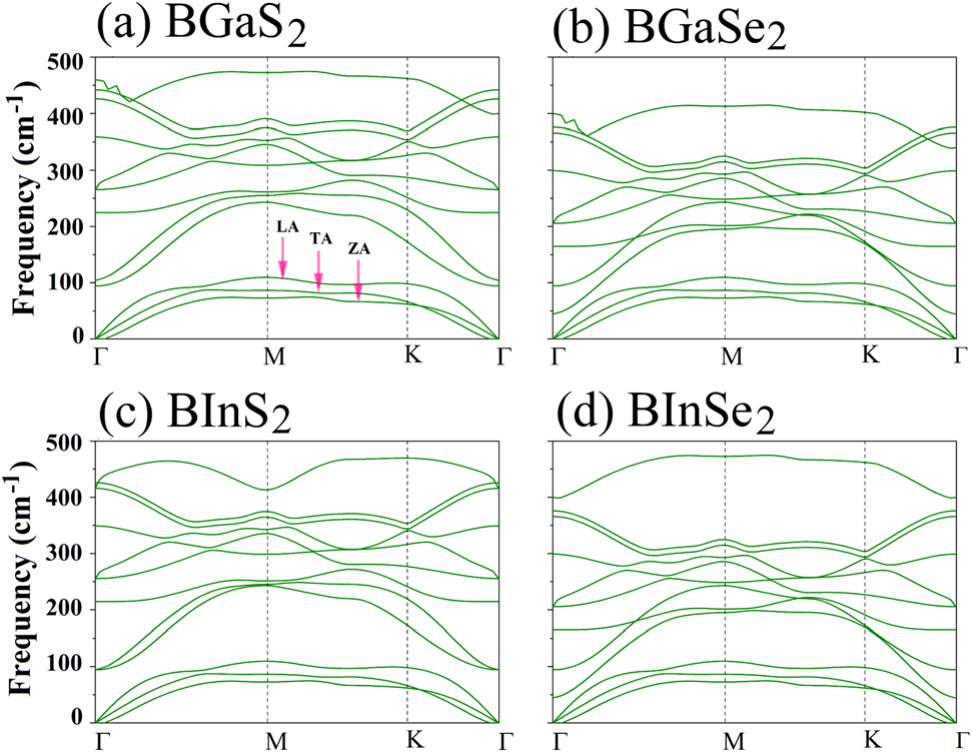
Calculated phonon dispersion curves along the high symmetry directions (*Γ*–*M*–*K*–*Γ*) in the Brillouin zone, for all of the 2D Janus monolayer BMX_2_ compounds considered.

Secondly, the dynamical stability of the BMX_2_ monolayers is investigated by analyzing their phonon dispersion curves, which were recorded along the high-symmetry directions (*Γ*–*M*–*K*–*Γ*) of the Brillouin zone. These curves are shown in Fig. 2. Given that the compounds have four atoms per unit cell, there are a total of 12 modes. The three lower frequency modes are acoustic modes, which include the in-plane longitudinal and transverse acoustic (LA and TA) modes, as well as the out-of-plane acoustic (ZA) mode. The general shapes of the phonon dispersions for the four monolayers are similar. The frequency values are all positive, with no imaginary phonon frequencies, indicating that the structures are dynamically stable and can exist as a 2D material. We now consider the mechanical stability of the Janus BMX_2_ monolayers.

### 3.2 Mechanical properties

The mechanical stability of each Janus monolayer is tested by finding its elastic constant (*C*_*ij*_).^37^ The elastic constants are key parameters that are proportional to the mechanical stability and the elastic properties of the material. The following equations were used to compute the in-plane elastic stiffness coefficients.^38^2
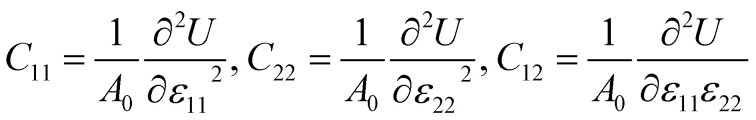
where *ε*_11_ and *ε*_22_ denote the *xy*-plane stress and *A*_0_ is equal to the crystal's equilibrium unit cell area, and *U* is the system's total energy. We examined *ε*_11_ and *ε*_22_ from −0.006 to +0.006 with a step size of 0.002. The atomic positions in the systems were relaxed under each strain, and we determined the relaxed-ion elastic stiffness coefficients C_11_, C_22_, and C_12_. The stability requirements may influence the mechanical stability criteria related to the crystal structure's elastic constants:^37^

3 (*C*_11_ − *C*_12_) > 0, *C*_11_ > 0, *C*_44_ > 0, and (*C*_11_ +2*C*_12_) > 0

As shown in Table 2, all Janus BMX_2_ compounds satisfy the calculated stability requirements. The following equations were used to compute the Young's modulus (*E*_*x*_) and Poisson's ratio (*ν*) using the elastic stiffness coefficients (*C*_*ij*_):^39^4
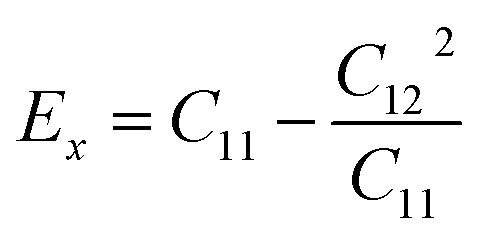
5
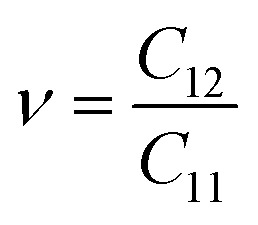


**Table 2:** Relaxed-ion elastic stiffness coefficients (*C*_*ij*_), Young's modulus (*E*_*x*_), and Poisson's ratio (*v*) for all the 2D Janus BMX_2_ compounds

Material	*C*_11_	*C*_22_	*C*_12_	*E*_*x*_ (N m^−1^)	*ν*
BGaS_2_	125.27	125.21	28.45	118.80	0.23
BGaSe_2_	120.72	120.68	27.12	114.62	0.23
BInS_2_	106.11	106.10	23.60	100.86	0.22
BInSe_2_	98.54	98.50	21.44	93.87	0.21

The stiffness is directly proportional to the Young's modulus. Therefore, more elasticity is based on having a higher Young's modulus. The Young's modulus ranges from 93.87 N m^−1^ (BInSe_2_) to 118.80 N m^−1^ (BGaS_2_), which are greater than those reported for BAlX_2_ (X = S, Se, Te) chalcogenide compounds.^40^ The gallium containing compounds have a greater Young's modulus than the indium containing ones. The dimensionless Poisson's ratio (*ν*) is an important metric in industrial production since it provides more direct information concerning adhesion strength than any other elastic constant. The calculated Poisson's ratios of the Janus compounds are between 0.21 and 0.23 which are comparable to those of well-known 2D materials like h-BN (0.23)^41^ and graphene (0.16).^42^ Importantly, the elastic constants of all four monolayers satisfy the Born criteria for mechanical stability.^43^ The elastic coefficients of BInSe_2_ are *C*_11_ = 98.54 N m^−1^ and *C*_12_ = 21.44 N m^−1^ and it is more flexible than other BMX_2_ compounds presented in this study.

### 3.3 Electronic properties

**Fig. 3:**
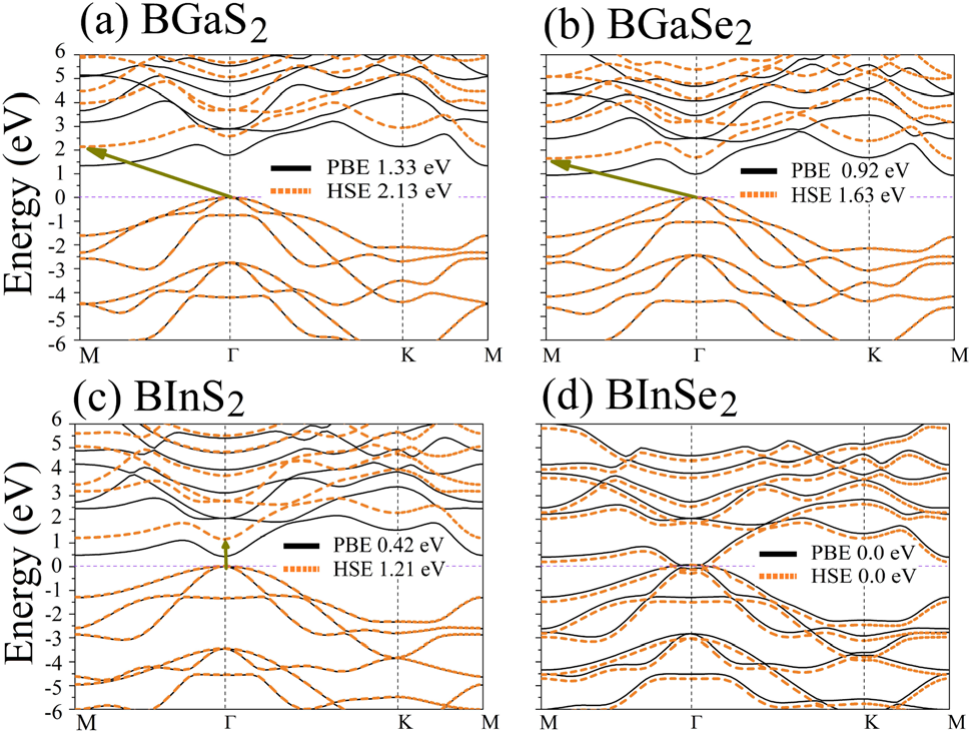
Band structures of the 2D Janus monolayer BMX_2_ compounds. Orange and black dotted lines correspond to results obtained using the HSE06 and PBE functionals, respectively. The dashed purple line represents the Fermi level, *E*_F_.

**Fig. 4:**
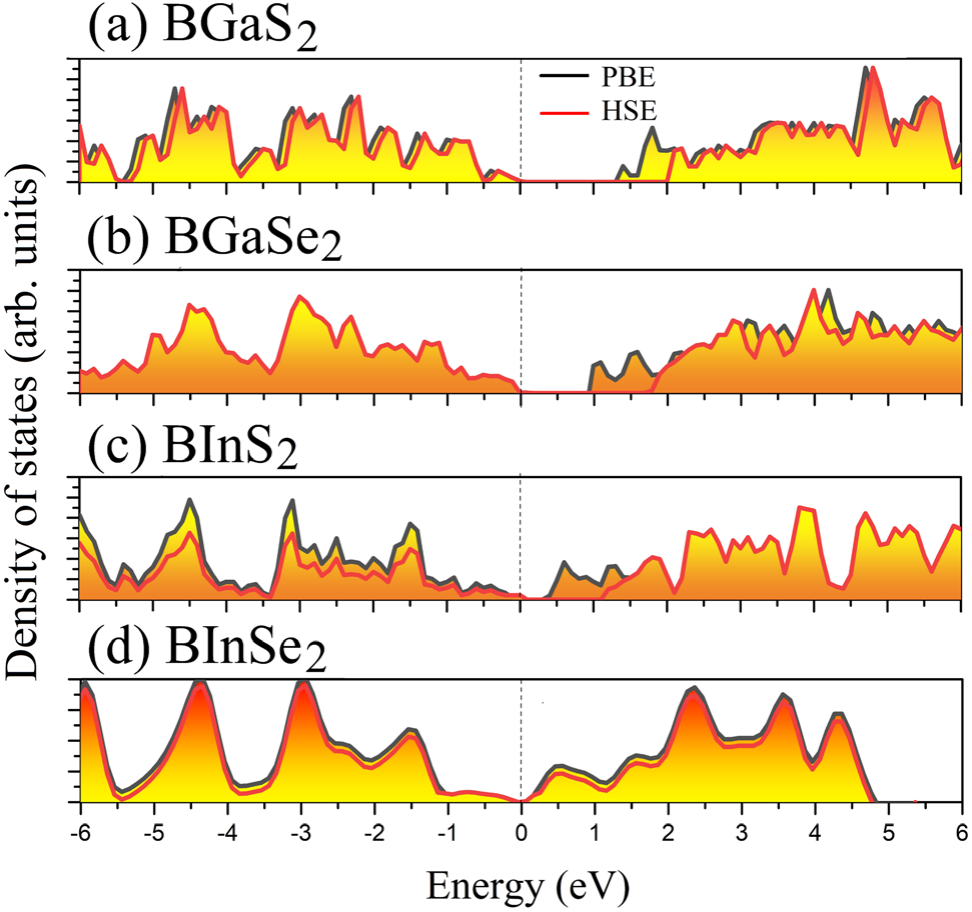
The total density of states (DOS) of the 2D Janus monolayers BMX_2_ compounds.

Optoelectronic applications rely on having particular electronic characteristics which include the type of band gap, the value of the band gap energy, and the density of states (DOS). Fig. 3 shows the band structures of the BMX_2_ compounds along the high-symmetry (*M*–*Γ*–*K*–*M*) direction as calculated by using both PBE and HSE06. When compared to the PBE functional, the hybrid HSE06 functional is known to produce a more reliable bandgap value since the PBE frequently leads to bandgap underestimation. The conduction band minimum (CBM) for both BGaS_2_ and BGaSe_2_ is located at the *K* point, while the valence band maximum (VBM) is located at the *Γ* point. Thus, both materials have an indirect bandgap. For BInS_2_, BGaS_2_ and BGaSe_2_ both the CBM and VBM are located at the *Γ* point, which indicates a direct bandgap. In particular, BInSe_2_ is shown to be a semimetal with zero bandgap at *Γ*. The calculated bandgap values of the BMX_2_ compounds using HSE06 (PBE) range from 0.42 (1.21) eV to 2.13 (1.33) eV, indicating that they are semiconductors with the exception of BInSe_2_.^44^ The corresponding total density of states (DOS) is shown in Fig. 4 and confirms the electronic type for each compound.

### 3.4 Optical properties

**Fig. 5:**
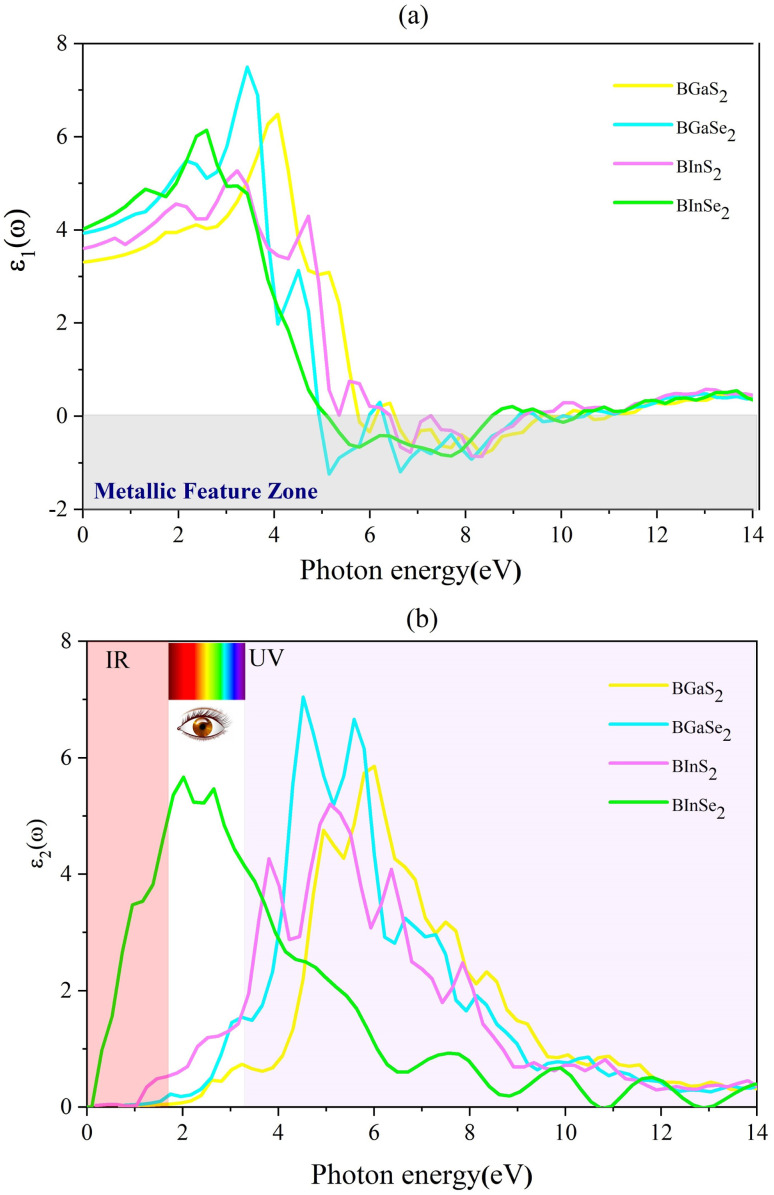
(a) The real (*ε*_1_) and (b) imaginary (*ε*_2_) parts of the dielectric function for the 2D Janus monolayer BMX_2_ compounds.

The optical absorption spectra are recorded from the frequency-dependent complex dielectric function^45^*ε*(*ω*) = *ε*_1_(*ω*) + i*ε*_2_(*ω*) by first-principles calculations within the random phase approximation (RPA).^46^*ε*_2_(*ω*), which represents the imaginary part of the dielectric function, is found by a summation over empty states from the Fermi golden rule:^47^6
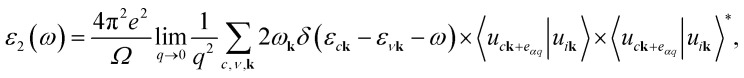
where *c* and *ν* represent the conduction and valence band states, respectively. *u*_*c***k**_ is the cell periodic part of the wavefunctions at the **k** point. The real part of the dielectric matrix *ε*_1_(*ω*) is found *via* the Kramers–Kronig^48^ transformation:7
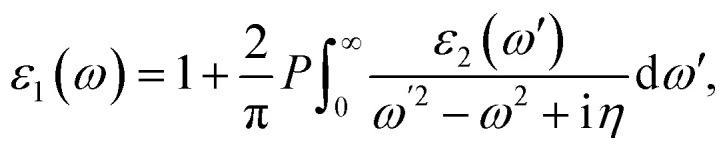
where *P* is the principal value of the integral. *ε*(*ω*) is split into 3 parts based on structural anisotropy: *xx*, *yy* and *zz* directions (*ε*_*xx*_ = *ε*_*yy*_) (in-plane) with intense in-plane peaks and (*ε*_*zz*_) out-of-plane with negligible peaks for the four monolayers. Fig. 5(a) and (b) display the dielectric function's real and imaginary components as a function of photon energy. For zero photon energy, the static dielectric constant is equal to the actual dielectric constant. For BInSe_2_, BGaSe_2_, BInSe_2_, and BGaSe_2_ monolayers, this intersection yields values of about 4.03, 3.93, 3.60, and 3.31, respectively, indicating that BInSe_2_ has a relatively high polarizability. Furthermore, the static dielectric constant at zero energy is compatible with InSe, InTe, and In_2_SeTe monolayers^49,50^ and is smaller compared to those of other two-dimensional materials.^51,52^ Interestingly, the BMX_2_ compounds have negative values in the range of 4.93–10.98 eV, indicating the metallic properties of our Janus monolayers in the UV region. For the imaginary part (Fig. 5(b)), there are several peaks for BInSe_2_ in the visible region at 2.01 eV and 2.65 eV and one peak at 7.55 eV in the ultraviolet range. It is noticed that for BInS_2_, the first peak is located at 1.22 eV, similar to the band gap of 1.21 eV, indicating that photoexcited electrons make a direct transition from the VBM to the CBM. Many peaks in the range of 3.8–6.5 eV can be seen due to the interband transitions for BGaS_2_ and BGaSe_2_. The maximum peak in the imaginary part shifted progressively to lower energies from 4.5 eV to 6 eV as S has been replaced by Se in BGaSe_2_. This coincides with the trend in the change of the band gap as dependent on composition.^53^ The wide absorption range of BInSe_2_, from infrared to ultraviolet light, suggests that BInSe_2_ is a good light harvesting material and suitable for optoelectronic applications and sensitive light sensors. Features in the range of 3.8–6.5 eV can be seen due to the interband transitions. The maximum peak of the imaginary part shifted progressively to low energies as S is replaced by Se in BGaSe_2_, which is consistent with the change found in the band gaps as above mentioned. The most significant feature is the broad absorption range of BInSe_2_, from infrared to ultraviolet light. This qualifies it as an optimal material for optoelectronic applications and sensitive light sensors.

### 3.5 Piezoelectric properties

We now investigate the piezoelectric properties of the BMX_2_ monolayers as utilized by Duerloo *et al.*^41^ We calculated the linear piezoelectric coefficients (*d*_*ijk*_) of the Janus group-III chalcogenide monolayers using the modern theory of polarization^43^ by monitoring the difference in polarization due to uniaxial stresses (*e*_*iik*_) as follows:8
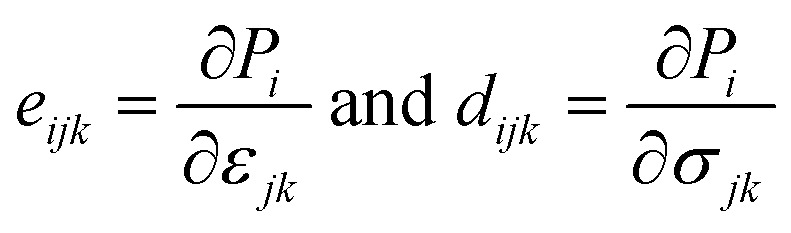
where *P*_*i*_ is the polarization vector; *e*_*ijk*_ and *r*_*ijk*_ are the strain and stress, respectively; *i*, *j*, and *k* indices correspond to the *x*, *y*, and *z* directions. The specific piezoelectric coefficients, as well as the out-of-plane coefficients, are determined for the Janus like-structures. These values are related to the elastic stiffness coefficients as follows:9
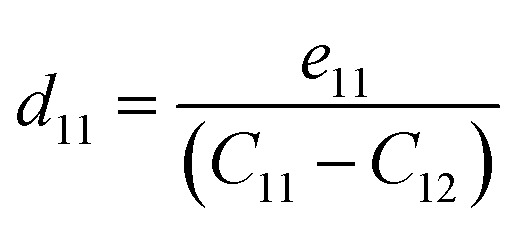
10
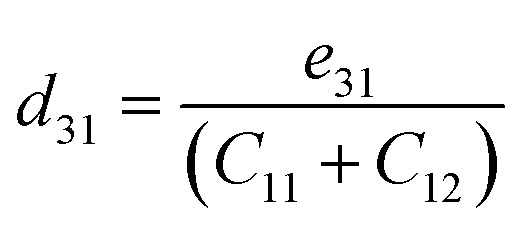


In our work, we consider the piezoelectric coefficients with the relaxed-ion method, because previous studies have shown that the clamped-ion method gives much larger values of *C*_*ij*_ than experimental values.^54,55^ As a result, we anticipate that the relaxed-ion method's piezoelectric coefficients will be more trustworthy.

**Fig. 6:**
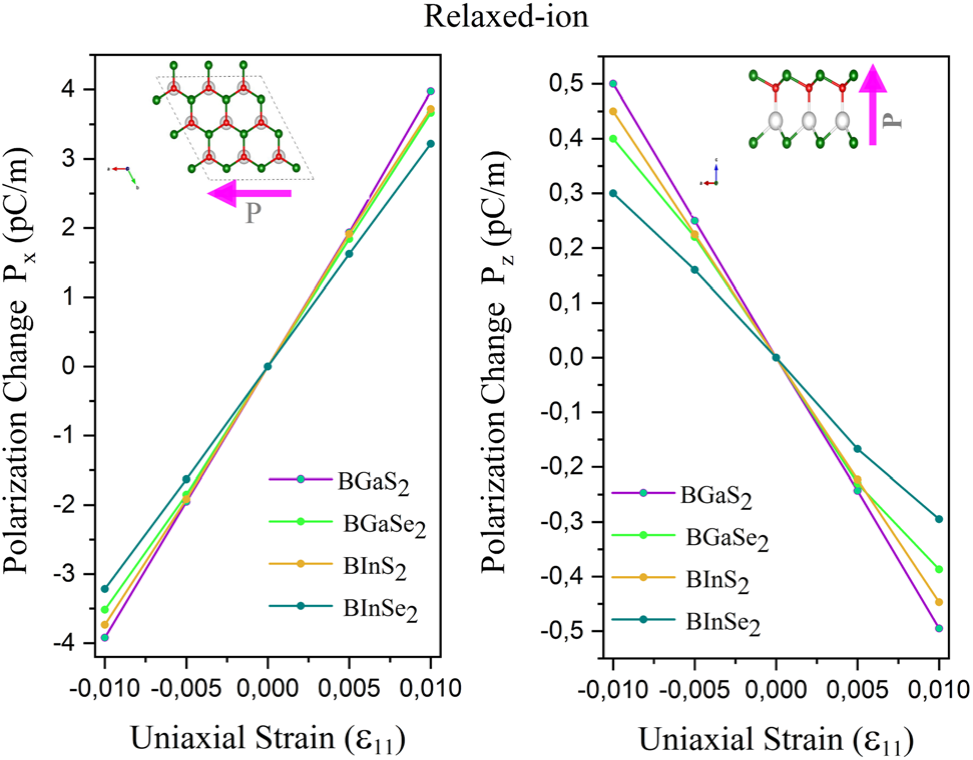
Polarization change as a function of strain for the 2D Janus monolayer BMX_2_ compounds.

The calculated *e*_*ij*_ and *d*_*ij*_ are summarized in Table 3 which are shown to be close to the reported values of various Janus monolayers in previous DFT calculations.^56,57^ This further validates our methodology. The choice of a heavier metal element M (from Ga to In) decreases the *d*_11_ and *e*_11_ values, which explains in this case BGaS_2_ having larger values than BInS_2_. We can see that such a piezoelectric response difference along the out-of-plane direction comes from the different electronegativities of each vertically stacked atom. The present compounds studied have out-of-plane piezoelectric coefficients, *d*_31_, in the range of 0.24–0.32 pm V^−1^, as shown in Table 3 and (Fig. 6 (right)). Interestingly, the BMX_2_ systems have high *d*_31_ (0.32–0.46 pm V^−1^) values, similar to GaInSe_2_ and GaInS_2_.^56^ The highest *d*_31_ value (0.32 pm V^−1^) occurs for the BGaSe_2_ monolayer, over three times the maximum value obtained for BAlSe_2_.^57^ The significant out-of-plane piezoelectric effect would provide these Janus monolayers with a diversity of piezoelectric features.

**Table 3:** Calculated piezoelectric coefficients, *e*_11_, *d*_11_, *e*_31_, and *d*_31_ of the studied BMX_2_ monolayers

Material	*e*_11_	*d*_11_	*e*_31_	*d*_31_
BGaS_2_	4.21	4.35	0.44	0.28
BGaSe_2_	3.83	4.10	0.48	0.32
BInS_2_	1.52	1.84	0.31	0.24
BInSe_2_	1.29	1.67	0.39	0.31
GaInS_2_ (ref. 56)	3.60	8.33	0.30	0.38
GaInSe_2_ (ref. 56)	0.86	3.19	0.29	0.46
AlBSe_2_ (ref. 57)	1.61	1.96	0.16	0.12

### 3.6 Ferroelectric properties

Ferroelectric 2D materials are a sub-class of materials with non-centrosymmetric structures that show spontaneous polarization in the absence of an electric field. As long as the temperature is below the Curie temperature (*T*_c_), ferroelectric materials exhibit spontaneous polarization. When the material is heated above *T*_c_, the polarization disappears due to atoms rearranging and the formation of a highly symmetric structure, and the material becomes paraelectric. Using first principles methods based on modern polarization theory^58^ in the three directions [*x*, *y*, *z*], we found that there is no in-plane polarization on the *x*-axis and *y*-axis, while there is very significant out-of-plane polarization along the *z*-axis (*P*_*z*_ > 0). The Janus monolayers have a large out-of-plane spontaneous electric polarization of 14.1 μC cm^−2^ to 16.2 μC cm^−2^ along the *z*-direction (Table 4).

**Table 4:** Out-of-plane spontaneous polarization values in the *z*-direction (*P*_*sz*_)

BMX_2_	*P*_*sz*_ (μC cm^−2^)
BGaS_2_	16.2
BGaSe_2_	15.3
BInS_2_	15.7
BInSe_2_	14.1
CuInP_2_S_6_ (ref. 59)	3.8
SnSe (ref. 60)	18.1
Sc_2_CO_2_ (ref. 61)	1.6
AgBiP_6_Se_6_ (ref. 62)	0.2

**Fig. 7:**
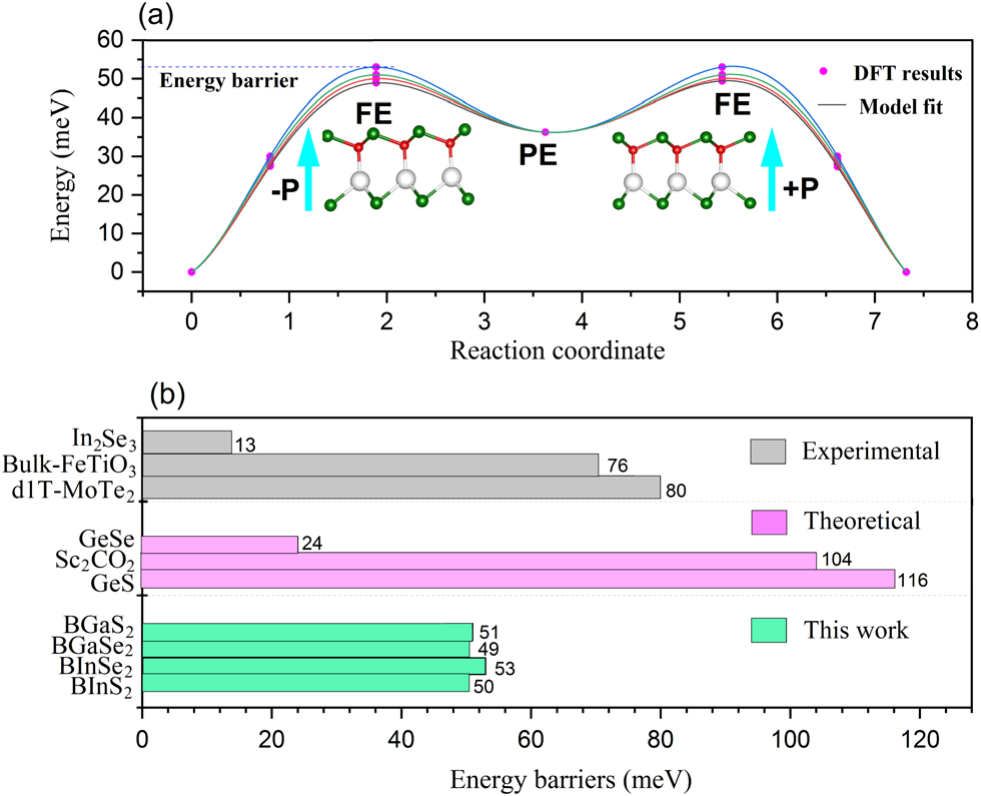
(a) The transition barrier for the 2D Janus monolayer BMX_2_ compounds. (b) Comparison between the predicted energy barriers of the BMX_2_ compounds and other theoretical and experimental energy barriers for the 2D ferroelectrics.

The polarization of the BMX_2_ compounds decreases as the chalcogen element changes from sulfur to selenium, whereas the electrical polarization *P* is positively correlated with the difference between the electronegativity of (B and M) atoms and the chalcogen elements. As the chalcogen element changes from S to Se, the reduced electronegativity results in a diminished polarization. Furthermore, the transition barrier is a vital property for evaluating a ferroelectric sensor's sensitivity. We constructed two structures that possess similar symmetry and total energy, but have opposing polarization orientations. The initial and final states of polarization are indicated as (−*P*) and (+*P*), respectively. We computed the transition barrier using the nudged elastic band (NEB) method.^63^ The results show that the energy barrier is around 50 meV, which is similar to that of the In_2_Se_2_ monolayer and a quarter of that of bulk ferroelectric PbTiO_3_ (200 meV).^64^ In Fig. 7(b), we also compare our result to other experimental^65–67^ and theoretical^60,61^ values. It is demonstrated in Fig. 7(a) that the polarization direction may be reversed by a negative electric field, which encourages the use of BMX_2_ compounds in high-density memory devices^68^ and low-consumption high-sensitivity ferroelectric devices.^69^

## 4 Conclusion

In conclusion, we explored the fundamental properties of Janus BMX_2_ monolayers by using first-principles calculations. Through analyzing both the phonon dispersion curves and elastic constants, it is found that all the studied Janus monolayers are dynamically and mechanically stable in the ground state. The calculations also showed how they are all more flexible than many other 2D materials. The calculation of electronic properties has shown how the monolayers (BGaS_2_ and BGaSe_2_), BInS_2_ and BInSe_2_ are (indirect), direct semiconductors and semi-metallic, respectively, ranging from 0 to 2 eV. We also studied the optical response of the four Janus monolayers, which are shown to have high absorption for light ranging from the infrared to the ultraviolet region of the spectrum. The BMX_2_ monolayers exhibited out-of-plane piezoelectric coefficients of up to 0.32 pm V^−1^, more than 2–3 times that of Janus aluminum-boron monochalcogenide monolayers. Our results not only systematically provide insight into the physical properties of the BMX_2_ monolayers but also represent an important step for further studies, especially for the potential of successfully synthesizing these 2D ternary boron Janus monolayers experimentally.

## Conflicts of interest

There are no conflicts to declare.

## Supplementary Material
